# Role of S1P/S1PR3 axis in release of CCL20 from human bronchial epithelial cells

**DOI:** 10.1371/journal.pone.0203211

**Published:** 2018-09-07

**Authors:** Yoshitaka Kawa, Tatsuya Nagano, Asuka Yoshizaki, Ryota Dokuni, Masahiro Katsurada, Tomomi Terashita, Yuichiro Yasuda, Kanoko Umezawa, Masatsugu Yamamoto, Hiroshi Kamiryo, Kazuyuki Kobayashi, Yoshihiro Nishimura

**Affiliations:** Division of Respiratory Medicine, Department of Internal Medicine, Kobe University Graduate School of Medicine, Kusunoki-cho, Chuo-ku, Kobe, Japan; Roswell Park Cancer Institute, UNITED STATES

## Abstract

**Background:**

Sphingosine kinase phosphorylates sphingosine to generate sphingosine 1 phosphate (S1P) following stimulation of the five plasma membrane G-protein-coupled receptors. The objective of this study is to clarify the role of S1P and its receptors (S1PRs), especially S1PR3 in airway epithelial cells.

**Methods:**

The effects of S1P on asthma-related genes expression were examined with the human bronchial epithelial cells BEAS-2B and Calu-3 using a transcriptome analysis and siRNA of S1PRs. To clarify the role of *CCL20* in the airway inflammation, BALB/c mice were immunized with ovalbumin (OVA) and subsequently challenged with an OVA-containing aerosol to induce asthma with or without intraperitoneal administration of anti-CCL20. Finally, the anti-inflammatory effect of VPC 23019, S1PR1/3 antagonist, in the OVA-induced asthma was examined.

**Results:**

S1P induced the expression of some asthma-related genes, such as *ADRB2*, *PTGER4*, and *CCL20*, in the bronchial epithelial cells. The knock-down of SIPR3 suppressed the expression of S1P-inducing *CCL20*. Anti-CCL20 antibody significantly attenuated the eosinophil numbers in the bronchoalveolar lavage fluid (*P<0*.*01*). Upon OVA challenge, VPC23019 exhibited substantially attenuated eosinophilic inflammation.

**Conclusions:**

S1P/S1PR3 pathways have a role in release of proinflammatory cytokines from bronchial epithelial cells. Our results suggest that S1P/S1PR3 may be a possible candidate for the treatment of bronchial asthma.

## Introduction

Sphingosine 1 phosphate (S1P) is a bioactive sphingolipid metabolite that regulates diverse biological responses such as proliferation, migration, cytoskeletal organization, adherent junction assembly and morphogenesis [1]. S1P is exported outside of cells by ATP-binding cassette-type or other transporters and extracellular S1P activates five G protein-coupled receptors, known as S1P receptors (S1PRs) 1–5 via autocrine and paracrine signaling [2].

S1PRs have diverse functions depending on the contexts. For example, S1PR1 has a role in lymphocyte trafficking, from the lymph nodes or thymus to the lymph or blood and in dendritic cell (DC) maturation [2]. In immune cells, the localizations of S1PRs are well-characterized, and the physiological role of the interaction between S1P and S1PRs in the immune cell function has been clarified using FTY720, which is a prodrug that is phosphorylated *in vivo* by sphingosine kinases (SPHK) 2 to biologically active FTY720-phosphate [3]. FTY720-phosphate binds to S1PRs except for S1PR2. Although it acts as a super agonist of S1PR1, FTY720-phophate leads to sustained S1PR1 internalization and lymphocyte sequestration [4]. The administration of FTY720 to the lung abrogates experimental asthma by inhibiting the migration of lung DCs to the regional lymph nodes [5]. In contrast, VPC23019 is an aryl amide-containing S1P analog that acts as an unselective competitive antagonist at both S1PR1 and S1PR3 [6]. Our laboratory previously showed that the administration of SPHK inhibitors prevented eosinophil inflammation [1].

A recent study showed that S1P elicits the gene expression of inflammatory cytokines, such as cyclooxygenase (COX)-2 mediated by phospholipase Cε (PLCε) in astrocytes [7]. We showed that PLCεis expressed in the bronchial epithelial cells (ECs) and has a role in asthma through upregulating the inflammatory cytokine production by the bronchial ECs in the elicitation phase [8]. In addition, we found that S1PR1-3 expressed on mouse airway ECs and S1PR2 had a role in nuclear factorκB (NF-κB) activation and CC chemokine ligand 3 (CCL) 3 production in the bronchial ECs [9]. However, JTE013, S1PR2 antagonist did not completely attenuate the ovalbumin (OVA)-induced airway inflammation. We think S1PR2 as well as S1PR1/3 regulate the chemokine production from lung structured cells.Therefore, this study builds upon our previous work and we would like to fully characterize the role of remaining S1P/S1PR3 axis in the bronchial ECs.

The aim of this study is to evaluate the role of S1P and S1PR3 in the airway ECs using human bronchial EC lines and experimental asthma mouse models.

## Materials and methods

### Reagents

VPC23019 (also known as BMS-345541; 401480, Calbiochem, Darmstadt, Germany) and JTE23019 (Cayman, Ann Arbor, MI) were commercially obtained. Sphingosine 1 phosphate (62570; Cayman chemical, Ann Arbor, MI, USA) and monoclonal rat anti-CCL 20 antibody (Clone # 114906, R & D Systems, Minneapolis, MN, USA) were used. S1P was supplied as a crystalline solid and was prepared by directly dissolving in basic buffers and 0.1% bovine serum albumin solutions according to the manufacturer's protocol.

### Bronchial epithelial cell cultures

Human bronchial EC lines BEAS-2B (CRL-9609) [10] and Calu-3 (HTB-55) [11] were purchased from ATCC (Manassas, VA, USA) and maintained in BEGM with BulletKit (#CC-3170; Lonza, Walkersville, MD, USA) and EMEM, respectively, in a humidified atmosphere containing 5% CO_2_ at 37°C. S1P induced interleukin-8 release via S1PR2 and nuclear factor κB in BEAS-2B cells [12]. In addition, the production of interleukin-8 was also observed in Calu-3 [13]. Therefore, these bronchial ECs were treated with or without 1 μM S1P for 2 h.

### RNA isolation and microarray

Total cellular RNA preparation from BEAS-2B and Calu-3 before and after S1P stimulation was performed as described [8, 9]. Total RNA labeled with Cy3 or Cy5 was hybridized to a 3D-Gene Human Oligo chip 25 k (Toray Industries Inc., Tokyo, Japan). Genes with Cy3/Cy5 normalized ratios greater than 2.0 were identified.

### siRNA and transfection

S1PR3#1 siRNA (s4455) and S1PR3#2 siRNA (s4454) were purchased from Life Technologies (Carlsbad, CA, USA). The negative control siRNA (sc37007) was purchased from Santa Cruz Biotechnology (Dallas, TX, USA). A total of 2×10^5^ cells were transfected with siRNA or control siRNA using the Lipofectamine^®^ RNA-iMAX Reagent (Life Technologies) in serum-free Opti-MEM^®^ Medium (Thermo Fisher Scientific, Waltham, MA, USA). After 24-h incubation, the cells were used for further experiments.

### Quantitative reverse transcription-polymerase chain reaction (qRT-PCR)

QRT-PCR was performed as described [8]. Relative human mRNA levels were calculated with the ΔΔCt method using the glyceraldehyde 3-phosphate dehydrogenase (*GAPDH*) mRNA as an internal control. The primers used in this study are as followed: 5’-GCACCGTCAAGGCTGAGAAC-3’ and 5’-ATGGTGGTGAAGACGCCAGT-3’ for *GAPDH* [14], 5’-CAGGTCTCCACTGCTGCC-3’ and 5’- CACTCAGCTCCAGGTCACT-3’ for *CCL3* [9], 5’- CCACCCAGAAGAAGAGCCTGAA-3’ and 5’-TGGACCCATGGGATGACTGTT-3’ for *TIMP2* [9], 5’-ATGACTTCCAAGCTGGCCGTG-3’ and 5’-TTATGAATTCTCAGCCCTCTTCAAAAACTTCTC-3’ for *IL-8* [15], 5’-TCAGGGAGGGCAGTATGTTC-3’ and 5’-GAGTAGAGGGGCAGGATGGT-3’ for *S1PR3* [16], and 5’-GACATAGCCCAAGAACAGAAA-3’ and 5’-GACAAGTCCAGTGAGGCACAA-3’ for *CCL20* [17].

### Western blot analysis

The detailed protocol for Western blotting has been described previously [14]. The indicating antibodies to the following proteins were used in this study: β-actin (#4967, Cell Signaling Technology, Danvers, MA, USA) and S1PR3 [EPR4540(2), Abcam, Cambridge, UK].

### Animals

Female BALB/c mice were purchased from CLEA Japan (Tokyo, Japan). Our research was approved by the Institutional Animal Care and Use Committee of Kobe University (Institutional Animal Care and Use Committee At Kusunoki and Myodani Campus Kobe University, Permit Numbers: P130610-R1, and P171009) and carried out according to the Kobe University Animal Experimentation Regulations. All surgery was performed under general anesthesia as follows: Mice were given dexmedetomidine (0.3 mg/kg; Maruishi Pharmaceutical, Osaka, Japan), midazolam (4 mg/kg; Astellas Pharma, Tokyo, Japan) and butorphanol tartrate (5 mg/kg; Meiji Seika Pharma, Tokyo, Japan) by intraperitoneal (i.p.) administration, and all efforts were made to minimize suffering.

### Induction of bronchial asthma

Six- to eight-week-old female BALB/c mice were sensitized with an i.p. injection of 10 μg of ovalbumin (Sigma-Aldrich, St. Louis, MO, USA) mixed with 1 mg of aluminum hydroxide in 0.5 ml of sterile phosphate-buffered saline (PBS) on days 0 and 7. On days 21 and 22, mice were placed in an acrylic chamber and exposed to aerosolized 1.0% OVA in sterile PBS for 30 min. Negative controls were injected and exposed to PBS on the same schedule.

### Experimental mouse models

To prepare the administered groups of mice, mice were given VPC23019 (1 μg/mouse), or anti-CCL20 antibody (20 μg/mouse) via i.p. injection 15 minutes before OVA inhalation on days 21 and 22.

### Bronchoalveolar lavage fluid (BALF)

Twenty-four hours after the final challenge of OVA inhalation, BAL was performed 3 times with 800 μl of PBS. Fluid was regularly recovered at 80%-90%. The BALF was collected and centrifuged at 1,500 × rpm for 5 min at 4°C. The pellet was suspended in PBS, and the leukocyte number was determined using hemocytometer. The leukocytes were then subjected to cytospin preparation and stained with Diff-Quick (Sysmex, Kobe, Japan). At least 200 leukocytes on each slide were subjected to differential counting of macrophages, neutrophils, lymphocytes and eosinophiols according to the standard morphological criteria [18].

### Statistical analysis

All data are expressed as the means ± standard error of the mean (SEM). We use an unpaired two-tailed Student’s *t*-test to compare the two different subjects. To test statistical differences between more than two groups, we use an analysis of variance (ANOVA). If *P* value was smaller than 0.05, the difference was considered to be statistically significant.

## Results

### Role of S1P in asthma-related gene expression in ECs

**Fig 1:**
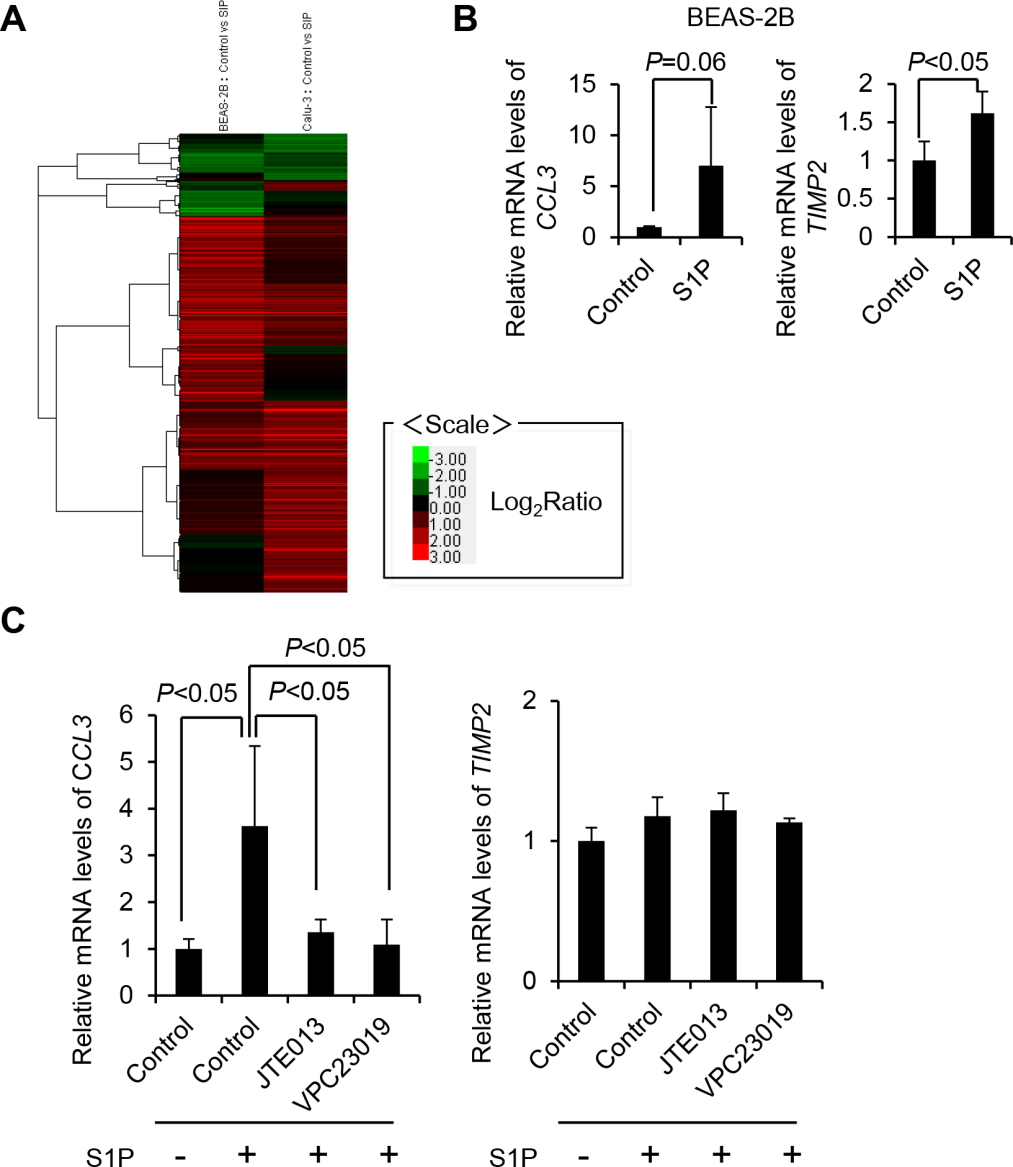
A hierarchical cluster analysis performed with Pearson’s correlation coefficient using log2 ratio data and qRT-PCR analysis of *CCL3* and *TIMP2*. BEAS-2B and Calu-3 were stimulated with or without S1P (1 μM) for 2 h. Total RNA labeled with Cy3 or Cy5 was hybridized to a 3D-Gene Human Oligo chip 25 k (n = 1). Genes with Cy3/Cy5 normalized ratios greater than 2.0 were identified. A total of 967 genes that were upregulated to more than 2-fold or downregulated to less than 2-fold in at least 1 pair were analyzed (A). Relative mRNA levels of *CCL3* and *TIMP2* were analyzed by qRT-PCR in BEAS-2 (B) and Calu-3 (C).

To evaluate the effect of S1P on the gene expression in ECs, we carried out a microarray analysis in two lines of ECs (GSE111746. BEAS-2B with or without S1P and Calu-3 with or without S1P). The expression of asthma-related genes is summarized in Table 1 [19, 20]. In contrast to S1P-untreated ECs, S1P-treated ECs expressed *ADRB2* and *PTGER4*. A hierarchical clustering analysis was performed using Pearson's correlation coefficient based on the log2 ratio data (Fig 1A and S1 Fig). We confirmed the increase of mRNA levels of *CCL3* and *TIMP2* in BEAS-2B cells (Fig 1B) and Calu-3 cells (Fig 1C). Intriguingly, not only JTE013 but also VPC23019 suppressed the increase of mRNA level of *CCL3*. Out of 967 genes that were up-regulated to more than 2-fold or down-regulated to less than 2-fold in at least 1 pair, only 14 genes were up-regulated 4-fold in both pairs (Table 2). Among these genes, *CCL20* was the gene most up-regulated in BEAS-2B. No previously reported asthma-related genes except for *CCL20* showed more than 4-fold upregulation or down-regulation in both cell lines [21]. CCL20 was reported to be secreted from bronchial ECs and regulates the recruitments of DCs which is critical for type 2 helper (T_H_2) responses in asthma [22]. The secretion of CCL20 was regulated by clusterin, an oxidative stress regulatory molecule [22]. In general, sphingolipids has nothing to do with clusterin, although sphingolipids also have a crucial role in the production of cytokine from epithelial cells [9]. Therefore, it is thought to be key question whether and how S1P regulates the production of CCL20 from bronchial ECs.

**Table 1:** The effects of S1P on the asthma-related gene expression in bronchial epithelial cells.

Gene	description	BEAS-2B	Calu-3
*ADRB2*	adrenoceptor beta 2	6.45	2.52
*LIFR*	leukemia inhibitory factor receptor alpha	0.89	1
*SCGB1A1*	secretoglobin family 1A member 1	1.13	1
*CTLA4*	cytotoxic T-lymphocyte associated protein 4	0.89	1.32
*ETV3*	ETS variant 3	1.52	1.37
*HAVCR2*	hepatitis A virus cellular receptor 2	0.71	1.8
*TLR1*	toll like receptor 1	N. D.	1.67
*GPRA(NPSR1)*	neuropeptide S receptor 1	N. D.	N. D.
*CCL11*	C-C motif chemokine ligand 11	0.92	1.04
*CCL24*	C-C motif chemokine ligand 24	0.96	1.01
*SFSWAP(SFRS8)*	splicing factor, suppressor of white-apricot family	0.71	1.08
*TNF*	tumor necrosis factor	N. D.	N. D.
*TLR2*	toll like receptor 2	1.01	0.98
*ADAMTS12*	ADAM metallopeptidase with thrombospondin type 1 motif 12	N. D.	N. D.
*TLR8*	toll like receptor 8	0.96	0.81
*TLR10*	toll like receptor 10	N. D.	N. D.
*PTGER4*	prostaglandin E receptor 4	8.53	4.64
*CCL2*	C-C motif chemokine ligand 2	1.37	N. D.
*PHF11*	PHD finger protein 11	1.1	1.34
*PRLR*	prolactin receptor	0.95	1.82
*TLR3*	toll like receptor 3	1.21	1.5
*SETDB2*	SET domain bifurcated 2	0.94	1.47
*CCL5*	C-C motif chemokine ligand 5	0.87	N. D.
*LTA*	lymphotoxin alpha	0.97	0.99
*IFNG*	interferon, gamma	2.42	N. D.
*GSTM1*	glutathione S-transferase mu 1	1.01	0.98
*ADAM33*	ADAM metallopeptidase domain 33	0.93	0.86
*ZFR*	zinc finger RNA binding protein	1.37	1.06
*TLR9*	toll like receptor 9	N. D.	N. D.
*PCDH1*	protocadherin 1	1.07	1.09
*NPR3*	natriuretic peptide receptor 3	0.83	N. D.
*IL4*	interleukin 4	0.46	0.83
*TLR7*	toll like receptor 7	1.13	0.65
*DPP10*	dipeptidyl peptidase like 10	0.95	1.05
*TGFB1*	transforming growth factor beta 1	1.2	0.87
*ORMDL3*	ORMDL sphingolipid biosynthesis regulator 3	1.03	1.03
*GSTP1*	glutathione S-transferase pi 1	0.98	1.04
*TLR8*	toll like receptor 8	N. D.	N. D.
*TLR5*	toll like receptor 5	0.8	2.88
*CHRM1*	cholinergic receptor, muscarinic 1	N. D.	N. D.
*CYFIP2*	cytoplasmic FMR1 interacting protein 2	1.19	1.09
*HLA-G*	major histocompatibility complex, class I, G	0.7	0.86
*MS4A2*	membrane spanning 4-domains A2	0.92	0.38
*STAT6*	signal transducer and activator of transcription 6, interleukin-4 induced	1.11	1.11
*IL13*	interleukin 13	0.92	0.92
*IL5*	interleukin 5	0.81	1.27
*RCBTB1*	RCC1 and BTB domain containing protein 1	0.87	0.9
*ETS2*	ETS proto-oncogene 2, transcription factor	1.34	2.7
*HAVCR1*	hepatitis A virus cellular receptor 1	N. D.	N. D.
*IL4R*	interleukin 4 receptor	1.39	1.25
*KITLG*	KIT ligand	1.07	1.21
*IL9*	interleukin 9	N. D.	N. D.
*MUC8*	mucin 8	0.97	1.21
*IL7R*	interleukin 7 receptor	1.31	2.12
*PTGDR*	prostaglandin D2 receptor (DP)	N. D.	N. D.
*LTC4S*	leukotriene C4 synthase	0.7	0.91
*CD14*	CD14 molecule	1.07	0.84
*TLR6*	toll like receptor 6	1.5	1.4
*NOS1*	nitric oxide synthase 1	N. D.	N. D.

The fold difference was obtained by dividing the mRNA level in S1P-treated ECs with that in S1P-untreated ECs.

**Table 2:** The S1P-inducing genes whose expression increased to four-fold or greater.

SYMBOL	description	BEAS-2B	Calu-3
*KRT34*	keratin 34, type I	8.84	5.56
*CCL20*	C-C motif chemokine ligand 20	53.65	7.33
*ZFP57*	ZFP57 zinc finger protein	11.27	6.13
*RNF39*	ring finger protein 39	5.73	6.95
*TAGLN*	transgelin	5.2	28.71
*EREG*	epiregulin	14.2	5.01
*PPP1R3B*	protein phosphatase 1 regulatory subunit 3B	6.43	10.33
*FST*	follistatin	5.47	9.47
*PTGS2*	prostaglandin-endoperoxide synthase 2	12.59	23.29
*EDN1*	endothelin 1	18.45	13.07
*SERPINB2*	serpin peptidase inhibitor, clade B (ovalbumin), member 2	25.01	59.43
*IL1B*	interleukin 1 beta	6.39	8.18
*GBP1*	guanylate binding protein 1	9	6.16
*CTGF*	connective tissue growth factor	20.12	12.83

Only 14 genes were up-regulated 4-fold in both pairs.

### Role of S1P/S1PR3 axis in release of CCL20 from bronchial ECs

**Fig 2:**
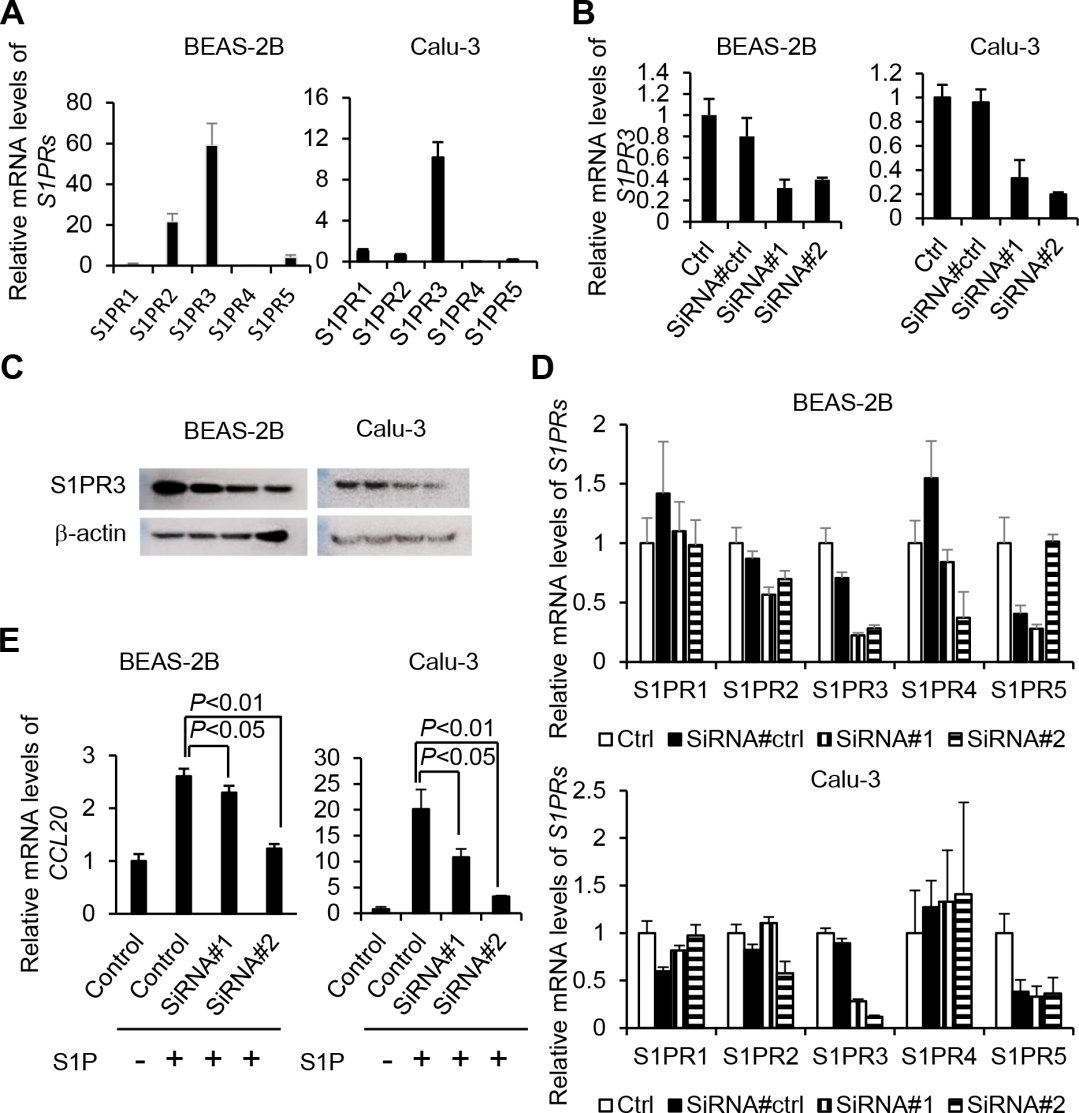
The assessment of role of S1PR3 in the bronchial epithelial cells. Relative mRNA levels of *S1PRs* in BEAS-2B and Calu-3 were analyzed by qRT-PCR (A). Gene expression knockdown of *S1PR3* was performed by the two pairs of siRNA, as described in the Materials and Methods. Data are expressed as the mean ± SEM (B). Protein levels of S1PR3 after the treatment of siRNA in BEAS-2B and Calu-3 were analyzed by western blot analyses (C). Relative mRNA levels of S1PRs after the treatment of siRNA in BEAS-2B and Calu-3 were analyzed by qRT-PCR (D). Relative mRNA levels of *CCL20* were analyzed by qRT-PCR in BEAS-2 and Calu-3 that had been treated with two pairs of siRNA of *S1PR3* and stimulated by PBS or 1 μM S1P for 2 h. Data are expressed as the mean ± SEM (E).

We analyzed the expression of *S1PRs* in the Calu-3 cells and confirmed the expression of *S1PR3* (Fig 2A). It could be possible that S1PR3 is more labile than S1PR2, and that is why the mRNA expression of *S1PR3* is higher than S1PR2. Therefore, we knocked down the *S1PR3* genes using siRNA. The efficacies of gene knockdown of *S1PR3* were about 80% (Fig 2B–2D). Ablation of *S1PR3* by siRNA significantly suppressed the gene expression of *CCL20* induced by S1P (*P*<0.01 to 0.05, Fig 2E). These results suggested that S1P induced *CCL20* expression via S1PR3.

### Attenuation of eosinophilic inflammation in the lung of VPC23019-treated mice

**Fig 3:**
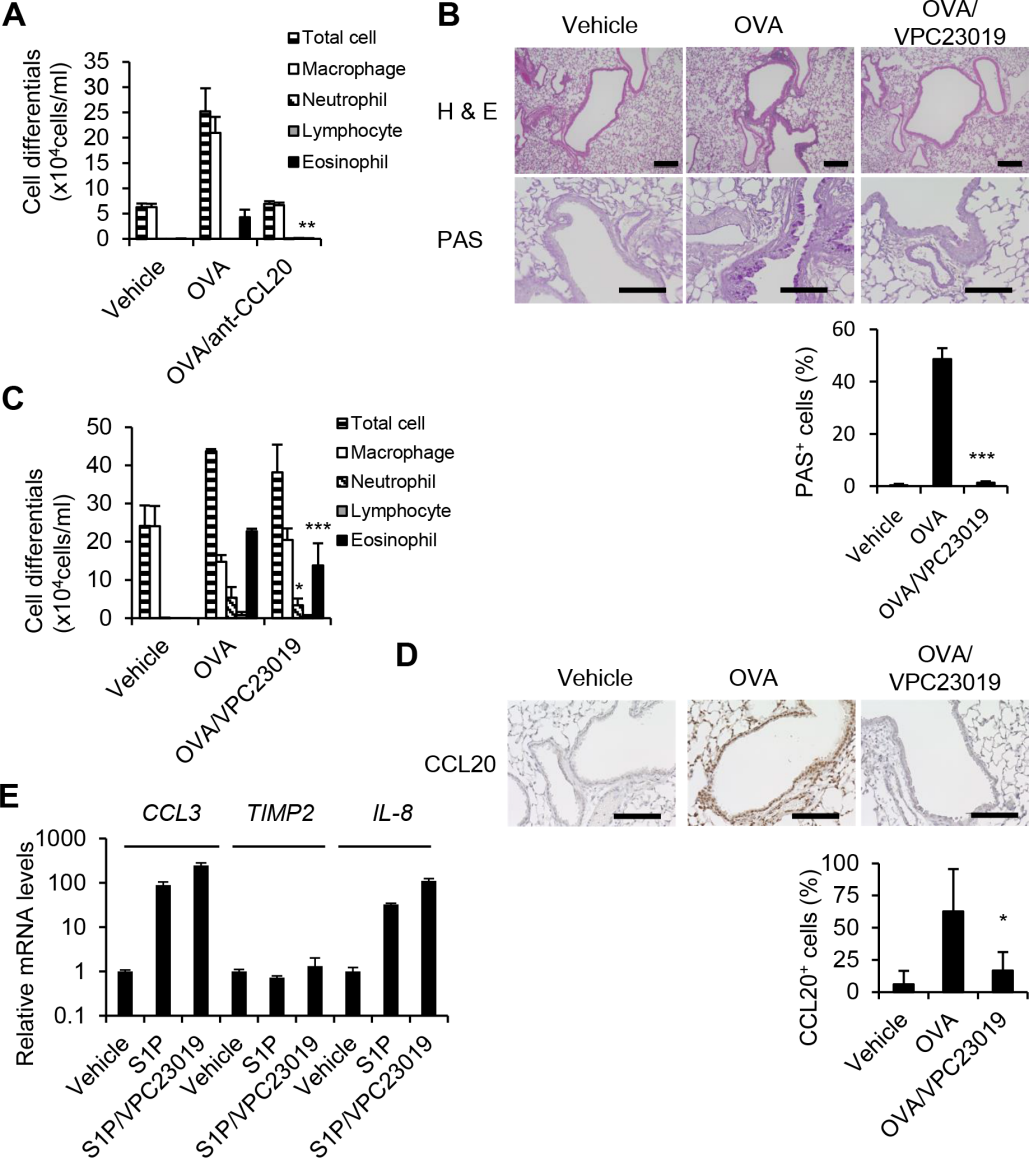
The analysis of the effect of VPC23019 in the experimental asthma mouse model. OVA-sensitized mice were administered PBS or anti-CCL20 antibody (20 μg/mouse) via i.p. injection before OVA inhalation on days 21 and 22. At day 23, BALF was collected, and inflammatory cells were analyzed. Data are expressed as the mean ± SEM obtained from three mice per group (A). Lungs and BALF were collected one day after the last challenge of the OVA-sensitized mice with the aerosol containing OVA or with PBS alone. Some mice received an intraperitoneal injection of 1 μg VPC23019 in a volume of 500 μl sterile PBS 30 minutes before each OVA exposure. Lung sections were subjected to H&E staining or PAS staining. *Bars*, 100 μm. PAS^+^ bronchial epithelial cells and total epithelial cells were counted on the specimens and the percentage of PAS^+^ epithelial cells was determined as 100 × (PAS^+^ cell number)/(total epithelial cell number) (%). Data are expressed as the mean ± SEM (n = 3) (B). The total inflammatory cells in the BALF were counted. For differential inflammatory cells counting, inflammatory cells were spun down and stained with Diff-Quick. Data are expressed as the mean ± SEM obtained for three mice in each group (C). Paraffin-embedded sections of the lung were stained with the antibody against CCL20 (*brown*) CCL20^+^ bronchial epithelial cells and total epithelial cells were counted on the specimens and the percentage of CCL20^+^ epithelial cells was determined as 100 × (CCL20^+^ cell number)/(total epithelial cell number) (%). Data are expressed as the mean ± SEM (n = 3) (D). BEAS-2B cells were treated with S1P (1 μM) and VPC23019 (10 μM) (B) for 3 h, and CCL3, TIMP2, and IL-8 gene expressions were analyzed by quantitative real-time RT-PCR. Data are expressed as the mean ± SEM (n = 3) (E). *, *P*<0.05 between three groups; **, *P*<0.01 between three groups; ***, *P*<0.001 between three groups.

To evaluate the role of CCL20 on bronchial asthma, we employed OVA-induced asthma mouse model. Twenty-four hours after the last OVA challenge, we performed BAL analyses and confirmed that the anti-CCL20 antibody reduced eosinophil percentage of total from 17% to 2% and significantly decreased eosinophil counts in the OVA-induced asthma mouse model (*P*<0.01, Fig 3A). This result suggested that CCL20 has a crucial role in the experimental asthma mouse model. To evaluate the effect of VPC23019, S1PR1 and S1PR3 antagonist, on experimental asthma, we carried out histologic analyses of the bronchus at 24 h after the last challenge. The accumulation of inflammatory cells around the bronchiole and mucin-producing epithelial cells positive for PAS staining was substantially reduced in VPC23019-treated mice (Fig 3B). Next, we performed a BAL examination for inflammatory cell counts using cytospin 24 h after the final OVA challenge using VPC23019). VPC23019 pretreatment significantly decreased the number of total cell and eosinophil cell compared vehicle pretreatment (*P*<0.05, Fig 3C). A similar trend was seen in the number of lymphocytes and neutrophils, but the difference was not statistically significant (*P*>0.05). Immunostaining demonstrated that S1P-induced CCL20 expression was suppressed by VPC23019 in lung structural cells (Fig 3D). On the oher hand, VPC23019 did not suppress the expression of mRNAs of *CCL3*, *TIMP2*, and *IL-8* (Fig 3E). These results suggested that VPC23019 attenuated the eosinophilic inflammation.

## Discussion

Sphingolipid metabolites have a role in the pathogenesis of asthma. S1P has been shown to promote airway hyper-reactivity through S1PR1 and S1PR3 and regulates immune cells, such as mast cells, eosinophils and DCs [23]. In the current study, we newly clarified the possible effects of S1P/S1PR3 axis on the expression of the CCL20 from the airway ECs and showed therapeutic effect of VPC23019 on airway eosinophilic inflammation.

The current study also showed that S1P induced *ADRB2* and *PTGER4* in the airway ECs. The β2-adrenoceptor gene (*ADRB2*) has genetic variability at amino acids 16 and 27 and correlates with some clinical features of bronchial asthma, including bronchial hyper-responsiveness [24, 25]. The Arg16 allele of *ADRB2* also has an important role in differential responses to β2-agonist therapy in bronchial asthma [26]. It is important to ensure consistency between humans and mice, which are one of the most commonly used species for modeling bronchial asthma. Similar to that of humans, the distribution of murine β2 adrenergic receptor (β2AR) subtypes is heterogeneous in lung [27]. B2AR is reportedly expressed in mouse tracheal epithelium (71%) and airway smooth muscle (ASM) (31%) [28, 29], and β2AR in bronchial smooth muscle plays an important role in bronchodilation in mice [30]. In addition, it was recently reported that β2AR signaling in airway ECs promotes eosinophilic inflammation, mucous metaplasia, and airway contractility [31]. In contrast, single nucleotide polymorphisms (SNPs) of prostaglandin E4 receptor (*PTGER4*) are reported to be associated with asthma symptoms [32]. The prostaglandin E (PGE) receptor 4 is expressed in a variety of tissues including lung, and has important roles in inflammation and immediate hypersensitivity. Recently, it was reported that S1P represses β2-adrenergic activity in human ASM cells by increasing cyclo-oxygenase (COX)-2-mediated PGE2 production [33]. Therefore, *ADRB2* and *PTGER4* may contribute to the S1P-induced experimental asthma mouse model. Table 1 show there is significant difference in the fold change of gene expression between BEAS-2B and Calu-3 cells. The kinetic difference among these cells in the [Ca^2+^]_i_ response which is induced by protease activated receptor 2 agonist peptide and Alternaria extract may be the cause of difference of cellular response [13].

In the current study, we focused on *CCL20*. A range of chemokines, including CCL20, CCL19, and CCL27, are primarily released by the bronchial ECs in response to stimulants, such as microbes or irritants [34]. These chemokines direct DC migration toward the epithelium and underlying mucosa through the ligands for CCR6, CCR7 and CCR10 [35]. Tumor necrosis factor-related apoptosis-inducing ligand (TRAIL) is abundantly expressed in the airway ECs of allergic mice and is related to the production of the chemokine CCL20. Inhibition of TRAIL is reported to impair the homing of myeloid DCs and T cells expressing CCR6 and CD4 to the airways, thereby suppressing several phenotypes of bronchial asthma, including T_H_2 cell cytokine release, inflammation, airway hyper-reactivity and the expression of the transcriptional activator STAT6 [36]. The current findings suggest that CCL20 has a crucial role in our experimental asthma model.

In conclusion, S1P/S1PR3 pathway has a role in release of CCL20, which has a role in experimental asthma mouse model, from bronchial ECs. Our results suggest that S1P/S1PR3 axis may be a possible candidate for the treatment of bronchial asthma.

## Supporting information

S1 Fig A hierarchical cluster analysis was performed with Pearson’s correlation coefficient using log2 ratio data. (XLSX)

## References

[1] Nishiuma T, Nishimura Y, Okada T, Kuramoto E, Kotani Y, Jahangeer S, et al Inhalation of sphingosine kinase inhibitor attenuates airway inflammation in asthmatic mouse model. Am J Physiol Lung Cell Mol Physiol. 2008;294:L1085–1093. 10.1152/ajplung.00445.2007 18359884

[2] Rivera J, Proia RL, Olivera A. The alliance of sphingosine-1-phosphate and its receptors in immunity. Nat Rev Immunol. 2008;8:753–763. 10.1038/nri2400 18787560 PMC2600775

[3] Ma B, Guckian KM, Lin EY, Lee WC, Scott D, Kumaravel G, et al Stereochemistry-activity relationship of orally active tetralin S1P agonist prodrugs. Bioorg Med Chem Lett. 2010;20:2264–2269. 10.1016/j.bmcl.2010.02.006 20188554 PMC2843818

[4] Mullershausen F, Zecri F, Cetin C, Billich A, Guerini D, Seuwen K. Persistent signaling induced by FTY720-phosphate is mediated by internalized S1P1 receptors. Nat Chem Biol. 2009;5:428–434. 10.1038/nchembio.173 19430484

[5] Idzko M, Hammad H, van Nimwegen M, Kool M, Müller T, Soullié T, et al Local application of FTY720 to the lung abrogates experimental asthma by altering dendritic cell function. J Clin Invest. 2006;116:2935–2944. 10.1172/JCI28295 17080194 PMC1626118

[6] Takabe K, Paugh SW, Milstien S, Spiegel S. "Inside-out" signaling of sphingosine-1-phosphate: therapeutic targets. Pharmacol Rev.2008;60:181–195. 10.1124/pr.107.07113 18552276 PMC2695666

[7] Dusaban SS, Purcell NH, Rockenstein E, Masliah E, Cho MK, Smrcka AV, et al Phospholipase Cε links G protein-coupled receptor activation to inflammatory astrocytic responses. Proc Natl Acad Sci U S A. 2013;110:3609–3614. 10.1073/pnas.1217355110 23401561 PMC3587233

[8] Nagano T, Edamatsu H, Kobayashi K, Takenaka N, Yamamoto M, Sasaki N, et al Phospholipase Cε, an effector of Ras and Rap small GTPases, is required for airway inflammatory response in a mouse model of bronchial asthma. PLoS One. 2014;9:e108373 10.1371/journal.pone.0108373 25269075 PMC4182471

[9] Terashita T, Kobayashi K, Nagano T, Kawa Y, Tamura D, Nakata K, et al Administration of JTE013 abrogates experimental asthma by regulating proinflammatory cytokine production from bronchial epithelial cells. Respir Res. 2016;17:146 10.1186/s12931-016-0465-x 27829417 PMC5103479

[10] Amstad P, Reddel RR, Pfeifer A, Malan-Shibley L, Mark GE 3rd, Harris CC. Neoplastic transformation of a human bronchial epithelial cell line by a recombinant retrovirus encoding viral Harvey ras. Mol Carcinog. 1988;1:151–160. 2855021 10.1002/mc.2940010303

[11] Lijnen HR, Zamarron C, Collen D. Characterization of the high-affinity interaction between human plasminogen and pro-urokinase. Eur J Biochem. 1985;150:141–144. 3926492 10.1111/j.1432-1033.1985.tb08999.x

[12] O'Sullivan MJ, Hirota N, Martin JG. Sphingosine 1-phosphate (S1P) induced interleukin-8 (IL-8) release is mediated by S1P receptor 2 and nuclear factor κB in BEAS-2B cells. PLoS One. 2014;9:e95566 10.1371/journal.pone.0095566 24743449 PMC3990666

[13] Matsuwaki Y, Wada K, White T, Moriyama H, Kita H. Alternaria fungus induces the production of GM-CSF, interleukin-6 and interleukin-8 and calcium signaling in human airway epithelium through protease-activated receptor 2. Int Arch Allergy Immunol. 2012;158:19–29. 10.1159/000337756 22627362 PMC3395436

[14] Hatakeyama Y, Kobayashi K, Nagano T, Tamura D, Yamamoto M, Tachihara M, et al Synergistic effects of pemetrexed and amrubicin in non-small cell lung cancer cell lines: Potential for combination therapy. Cancer Lett 2014;343:74–79. 10.1016/j.canlet.2013.09.019 24139969

[15] de Waal Malefyt R, Abrams J, Bennett B, Figdor CG, de Vries JE. Interleukin 10(IL-10) inhibits cytokine synthesis by human monocytes: an autoregulatory role of IL-10 produced by monocytes. J Exp Med. 1991;174:1209–1220. 1940799 10.1084/jem.174.5.1209 PMC2119001

[16] Estrada R, Zeng Q, Lu H, Sarojini H, Lee JF, Mathis SP, et al Up-regulating sphingosine 1-phosphate receptor-2 signaling impairs chemotactic, wound-healing, and morphogenetic responses in senescent endothelial cells. J Biol Chem. 2008;283:30363–30375. 10.1074/jbc.M804392200 18765664 PMC2573088

[17] Lin SZ, Chen KJ, Xu ZY, Chen H, Zhou L, Xie HY, et al Prediction of recurrence and survival in hepatocellular carcinoma based on two Cox models mainly determined by FoxP3^+^ regulatory T cells. Cancer Prev Res (Phila). 2013;6:594–602. 23599540 10.1158/1940-6207.CAPR-12-0379

[18] van Rijt LS, Kuipers H, Vos N, Hijdra D, Hoogsteden HC, Lambrecht BN. A rapid flow cytometric method for determining the cellular composition of bronchoalveolar lavage fluid cells in mouse models of asthma. J Immunol Methods. 2004;288:111–121. 10.1016/j.jim.2004.03.004 15183090

[19] Holgate ST. The airway epithelium is central to the pathogenesis of asthma. Allergol Int. 2008;57:1–10. 10.2332/allergolint.R-07-154 18209502

[20] Szalai C, Ungvári I, Pelyhe L, Tölgyesi G, Falus A. Asthma from a pharmacogenomic point of view. Br J Pharmacol. 2008;153:1602–1614. 10.1038/bjp.2008.55 18311188 PMC2438267

[21] Holgate ST. Innate and adaptive immune responses in asthma. Nat Med. 2012;18:673–683. 10.1038/nm.2731 22561831

[22] Hong GH, Kwon HS, Moon KA, Park SY, Park S, Lee KY, et al Clusterin Modulates Allergic Airway Inflammation by Attenuating CCL20-Mediated Dendritic Cell Recruitment. J Immunol. 2016;196:2021–2030. 10.4049/jimmunol.1500747 26826245

[23] Maceyka M, Spiegel S. Sphingolipid metabolites in inflammatory disease. Nature. 2014;510:58–67. 10.1038/nature13475 24899305 PMC4320971

[24] D'amato M, Vitiani LR, Petrelli G, Ferrigno L, di Pietro A, Trezza R, et al Association of persistent bronchial hyperresponsiveness with beta2-adrenoceptor (ADRB2) haplotypes. A population study. Am J Respir Crit Care Med. 1998;158:1968–1973. 10.1164/ajrccm.158.6.9804126 9847294

[25] Liggett SB. Polymorphisms of adrenergic receptors: variations on a theme. Assay Drug Dev Technol. 2003;1:317–326. 10.1089/15406580360545134 15090197

[26] Hall IP, Blakey JD, Al Balushi KA, Wheatley A, Sayers I, Pembrey ME, et al Beta2-adrenoceptor polymorphisms and asthma from childhood to middle age in the British 1958 birth cohort: a genetic association study. Lancet. 2006;368:771–779. 10.1016/S0140-6736(06)69287-8 16935688

[27] Hegde A, Strachan RT, Walker JK. Quantification of beta adrenergic receptor subtypes in beta-arrestin knockout mouse airways. PLoS One. 2015;10:e0116458 10.1371/journal.pone.0116458 25658948 PMC4319755

[28] Henry PJ, Goldie RG. Beta 1-adrenoceptors mediate smooth muscle relaxation in mouse isolated trachea. Br J Pharmacol. 1990;99:131–135. 2158831 10.1111/j.1476-5381.1990.tb14666.x PMC1917490

[29] Henry PJ, Rigby PJ, Goldie RG. Distribution of beta 1- and beta 2-adrenoceptors in mouse trachea and lung: a quantitative autoradiographic study. Br J Pharmacol. 1990;99:136–144. 1970491 10.1111/j.1476-5381.1990.tb14667.x PMC1917506

[30] Lin R, Degan S, Theriot BS, Fischer BM, Strachan RT, Liang J, et al Chronic treatment in vivo with β-adrenoceptor agonists induces dysfunction of airway β(2) -adrenoceptors and exacerbates lung inflammation in mice. Br J Pharmacol. 2012;165:2365–2377. 10.1111/j.1476-5381.2011.01725.x 22013997 PMC3413869

[31] Nguyen LP, Al-Sawalha NA, Parra S, Pokkunuri I, Omoluabi O, Okulate AA, et al β2-Adrenoceptor signaling in airway epithelial cells promotes eosinophilic inflammation, mucous metaplasia, and airway contractility. Proc Natl Acad Sci U S A. 2017;114:E9163–E9171. 10.1073/pnas.1710196114 29073113 PMC5664525

[32] Kurz T, Hoffjan S, Hayes MG, Schneider D, Nicolae R, Heinzmann A, et al Fine mapping and positional candidate studies on chromosome 5p13 identify multiple asthma susceptibility loci. J Allergy Clin Immunol. 2006;118:396–402. 10.1016/j.jaci.2006.04.036 16890764

[33] Rumzhum NN, Rahman MM, Oliver BG, Ammit AJ. Effect of Sphingosine 1-Phosphate on Cyclo-Oxygenase-2 Expression, Prostaglandin E2 Secretion, and β2-Adrenergic Receptor Desensitization. Am J Respir Cell Mol Biol. 2016;54:128–135. 10.1165/rcmb.2014-0443OC 26098693

[34] McWilliam AS, Nelson D, Thomas JA, Holt PG. Rapid dendritic cell recruitment is a hallmark of the acute inflammatory response at mucosal surfaces. J Exp Med. 1994;179:1331–1336. 8145044 10.1084/jem.179.4.1331 PMC2191461

[35] Hammad H, Plantinga M, Deswarte K, Pouliot P, Willart MA, Kool M, et al Inflammatory dendritic cells-not basophils-are necessary and sufficient for induction of Th2 immunity to inhaled house dust mite allergen. J Exp Med. 2010;207:2097–2111. 10.1084/jem.20101563 20819925 PMC2947072

[36] Weckmann M, Collison A, Simpson JL, Kopp MV, Wark PA, Smyth MJ, et al Critical link between TRAIL and CCL20 for the activation of TH2 cells and the expression of allergic airway disease. Nat Med. 2007;13:1308–1315. 10.1038/nm1660 17934471

